# Epidemiological and clinical risk factors related to severe COVID-19 in Iran: a multi-center study

**DOI:** 10.1186/s12879-022-07165-0

**Published:** 2022-02-23

**Authors:** Seyed Mohammad Hashemi-Shahri, Seyed Mohammad Nasiraldin Tabatabaei, Alireza Ansari-Moghaddam, Mahdi Mohammadi, Hassan Okati-Aliabad, Seyed Mehdi Tabatabaei, Hossein Ansari, Mohammadhadi Abbasi, Khodadad Sheikhzadeh, Mehdi Zanganeh Baygi, Majid Sartipi, Sharareh Sanei-Sistani, Ali Reza Salimi Khorashad, Fatemeh Ansari-Moghadam, Neda Torab, Tahereh Khalili, Ghasem Miri-Aliabad

**Affiliations:** 1 Research Center for Infectious Diseases and Tropical Medicine, Zahedan University of Medical Sciences, Zahedan, Iran; 2 Department of Anesthesiology, Zahedan University of Medical Sciences, Zahedan, Iran; 3 Health Promotion Research Center, Zahedan University of Medical Sciences Campus, Hesabi Square, Zahedan, Iran; 4 Department of Epidemiology and Biostatistics, School of Health, Zahedan University of Medical Sciences, Zahedan, Iran; 5 Department of Radiology, Zahedan University of Medical Sciences, Zahedan, Iran; 6 Department of Biochemistry, School of Medicine, Zahedan University of Medical Sciences, Zahedan, Iran; 7 Children and Adolescent Research Center, Zahedan University of Medical Sciences, Zahedan, Iran

**Keywords:** COVID-19, Patients, Risk factors, Severity

## Abstract

**Background:**

Iran was one of the first countries to be affected by COVID-19. Identifying factors associated with the severity of COVID-19 is effective in disease management. This study investigated the epidemiological and clinical features and factors related to the severity of COVID-19 in one of the less privileged areas in Iran.

**Methods:**

In a multi-center study, all patients admitted to Zahedan University of Medical Sciences hospitals in southeastern Iran were investigated from February 29 to April 31, 2020. Demographic, epidemiological, and clinical data of patients were extracted from medical records. Bivariate and multivariate logistic regression models were used to explore the risk factors associated with the severity of COVID-19.

**Results:**

Among the 413 patients, 55.5% were male, and 145 (35.10%) were in a severe condition at admission time. Multivariate analysis showed that the adjusted odds of the disease severity increased in patients with older age (OR 2.27; 95% CI 1.41–3.65), substance abuse (OR 2.49; 95% CI 1.14–5.43), having one underlying disease (OR 1.52; 95% CI 0.90–2.55), having two underlying disease (OR 2.31; 95% CI 1.19–4.50), and having three or more underlying disease (OR 2.60; 95% CI 1.19–5.66).

**Conclusions:**

COVID-19 was more severe in older patients, patients with a history of substance abuse, and patients with the underlying disease. Understanding the factors affecting the disease severity can help the clinical management of COVID-19, especially in less privileged areas where fewer resources are available.

## Background

The SARS-CoV-2 has caused a global outbreak and is a significant public health issue worldwide. This virus has spread dramatically since its emergence in China at the end of 2019 [1]. On February 11, 2020, the World Health Organization (WHO) announced a new name for the epidemic disease caused by novel coronavirus: COVID-19 [2]. COVID-19 pandemic was declared by the WHO worldwide. Until the writing of this manuscript, it was most widespread in the United States of America, Brazil, India, Russian Federation, South Africa, Peru, Mexico, Colombia, Chile, and Iran, respectively. In the meantime, 249 (5.74%) death of all 369,911 confirmed patients in Iran occurred [3]. However, there is no comprehensive report regarding risk factors related to severe COVID-19 disease in Iran.

Regarding the risk factors for the severity of COVID-19, three pooled studies in China showed that 20.1% of patients developed acute respiratory distress syndrome. Furthermore, 25.9% required ICU admission, 8.3% invasive mechanical ventilation, and 3.2% extracorporeal membrane oxygenation for refractory hypoxemia [4–6]. However, the ratio for severe COVID-19 patients is dissimilar in different countries [1, 7].

There is evidence that hypertension, respiratory disease, cardiovascular disease increase the risk of severe COVID-19 [8–10], and men are at higher risk [11]. However, it seems that health records are often incomplete or inaccurate [12], and it is expected that the severity pattern of COVID-19 and its risk factors will change soon. Morbidity to severe COVID-19 will increase the risk of death and consequently impose more expense on the health system. Therefore, it gives the impression that the estimation of risk factors for severe COVID-19 could be helpful for physicians to manage the patient treatment and effectively prioritize resources for patients with the highest risk [13], especially in deprived regions. Evidence shows that individuals with lower socioeconomic status during the COVID-19 pandemic may be more vulnerable and neglected, possibly because of disease burden, poor health literacy and access to healthcare [14]. Social factors that either lead to barriers to timely access to care or create a situation in which patients consider the delay in care the most reasonable option influence the disparities in outcomes between COVID-19 patients [15].

To our knowledge, no previous report has been published from patients with severe conditions and their characteristics in less privileged areas of Iran. Sistan and Baluchistan province is located in the southeast of Iran and has common borders with Pakistan and Afghanistan. This province has the lowest position in the development indices of the provinces of Iran. Zahedan is the capital of Sistan and Baluchestan province [16]. It is hypothesized that the characteristics and risk factors for COVID-19 are different in this area. Therefore, we aimed to determine the potential risk factors of severe COVID-19 patients and describe patients’ epidemiological and clinical characteristics during hospitalization. As the first study of this region, the results could also provide the basis for comparison in future epidemiological studies.

## Methods

### Study design and population

This study was carried out on 413 COVID-19 patients in all hospitals affiliated with Zahedan University of Medical Sciences, southeast of Iran. All patients diagnosed with COVID-19 according to WHO interim guidance [17] between February 29_,_ 2020, and April 31, 2020, were included in the study. These hospitals were referral for the transfer of patients with COVID-19 from other medical centers in Zahedan. Therefore, the current study considered all adult inpatients hospitalized for COVID-19 during the mentioned time.

### Evaluation of clinical results

According to the WHO clinical management of COVID-19 guideline, symptomatic patients without evidence of viral pneumonia or hypoxia were classified as a mild disease. Patients with clinical signs of pneumonia but no signs of severe pneumonia were grouped as a moderate disease. Patients with oxygen support were classified as a severe disease, and those with further complications were classified as a critically ill disease [17].

### Data collection

Epidemiological and demographic characteristics and clinical data (symptoms, underlying disease, treatments, complications after admission, and outcomes) were extracted from medical records. In addition, the complementary data were collected by interviews with patients or their companions through two well-informed infectious disease residents and one anesthesia resident during hospitalization and referring to health centers during the early stage of the disease. Exposure history was defined as exposure to people with confirmed COVID-19 infection. Special and sub-special physicians approved all clinical data, symptoms, and signs based on standard definitions. Two researchers checked all data.

COVID-19 detection in respiratory specimens by RT-PCR methods was supervised by the center for disease control and prevention at the ministry of health in Iran. At the commence of the epidemic, the throat-swab specimens obtained from COVID-19 suspects were sent to the reference laboratory at Tehran University of Medical Sciences (school of public health) and Pasteur Institute of Iran. From March 11_,_ 2020, the specimens were examined in a laboratory launching for COVID-19 detection in Zahedan city. The throat-swab samples were obtained from patients with fever, cough, and dyspnea symptoms. A confirmed case was defined as a suspected case with a positive test of RT-PCR assay on respiratory specimens.

### Statistical analysis

The absolute frequency and percent were used to describe the categorical variables. The mean and standard deviation was used to summarize continuous variables. Differences between severe and non-severe patients regarding epidemiological and clinical factors and underlying diseases were compared using independent samples t-test, chi-square test, Fisher’s exact test. Bivariate and multivariate logistic regression models were used to explore the risk factors associated with disease severity. The logistic regression was chosen for multivariate analysis based on previous findings and clinical constraints considering the collinearity before fitting the model. We excluded variables from the bivariate analysis if their between-group differences were not significant, accuracy was unconfirmed, and the number of events was too small to calculate odds ratios. The data were analyzed in Stata.16, and the significance level was considered as 0.05.

## Results

Of 413 patients admitted to hospitals, 55.5% were male, 38.5% had aged more than 50. The mean age of the patients was 45.05 ± 17.38, ranging from 10 to 92 years. Regarding epidemiological characteristics, 9% and 3.9% of patients reported drug use and cigarette smoking, respectively. Patients who traveled to high-risk areas within 14 days before the onset of symptoms were 13.6%. Close contact with a respiratory patient in a medical center, family, and workplace was 5.9%, 9.2%, and 2.7%, respectively. About 4% of patients reported a history of close contact with a COVID19 patient within 14 days before the onset of symptoms. Among patients, 41.4% used mask protection, and 54.7% personally went to a medical center. At admission, 145 (35.10%) patients were in severe and 268 (64.90%) non-severe conditions. There was a significant relationship between age and severity of COVID-19. The odds of severe disease were 3.38 times greater in patients aged more than 50 years, and it was 2.09 times greater in substance abuse cases. Patients referred to the hospital by ambulance (OR 3.53) were more likely to be in a severe condition of COVID-19 compared to those who went to the hospital personally. Although wearing a mask decreased the odds of severity (OR 0.38), there was no significant relationship between the mask protection and the disease severity. There were 16 (3.9%) cigarette smokers, and smoking was not the predictor of severity among COVID-19 patients (Table 1).

**Table 1:** Demographic and epidemiological characteristics of severe COVID-19 patients in the southeast Iran

Demographics and epidemiological characteristics	Total	Severe	OR (95% CI)
	N (%)	N (%)	
Age (year)			
Less/equal 50	254 (61.5)	62 (24.4)	1
More than 50	159 (38.5)	83 (52.2)	3.38 (2.22,5.16)*
Sex			
Female	184 (44.5)	65 (35.3)	1
Male	229 (55.5)	80 (34.9)	0.98 (0.66,1.48)^NS^
Cigarette smoking			
No	397 (96.1)	138 (34.8)	1
Yes	16 (3.9)	7 (43.8)	1.46 (0.53,4.00) ^NS^
Substance abuse			
No	376 (91)	126 (33.5)	1
Yes	37 (9)	19 (51.4)	2.09 (1.06,4.13)*
Travel to high-risk areas			
No	357 (86.4)	136 (38.1)	1
Yes	56 (13.6)	9 (16.1)	0.31 (0.14,0.65)*
Go to the medical care center within 14 days before the onset of symptoms			
No	362 (87.6)	122 (33.7)	1
Yes	51 (12.4)	23 (45.1)	1.62 (0.89,2.92) ^NS^
Close contact with a respiratory patient within 14 days before the onset of symptoms			
No	365 (88.3)	127 (34.8)	1
Yes	48 (11.7)	18 (37.5)	1.12 (0.60,2.10) ^NS^
Close contact with a respiratory patient within 14 days before the onset of symptoms in medical centers			
No	389 (94.1)	133 (34.2)	1
Yes	24 (5.9)	12 (50.0)	1.93 (0.84,4.40) ^NS^
Close contact with a respiratory patient within 14 days before the onset of symptoms in the family			
No	375 (90.8)	130 (34.7)	1
Yes	38 (9.2)	15 (39.5)	1.23 (0.62,2.44) ^NS^
Close contact with a respiratory patient within 14 days before the onset of symptoms in the workplace			
No	402 (97.3)	142 (35.3)	1
Yes	11 (2.7)	3 (27.3)	0.69 (0.18,2.63) ^NS^
History of close contact with a COVID19 patient within 14 days before the onset of symptoms			
No	397 (96.1)	137 (34.5)	1
Yes	16 (3.9)	8 (50.0)	1.90 (0.70,5.17) ^NS^
Using Mask protection			
No	242 (58.6)	106 (43.8)	1
Yes	171 (41.4)	39 (22.8)	0.38 (0.25,0.59) ^NS^
How transferred to a medical center			
Personally	223 (54.4)	54 (24.2)	1
By others	104 (25.3)	44 (42.3)	2.30 (1.40,3.77)*
By ambulance	83 (20.3)	44 (53.0)	3.53 (2.08,5.99)*
Distance protection			
No	404 (97.8)	142 (35.1)	1
Yes	9 (2.2)	3 (33.3)	0.92 (0.23,3.74) ^NS^
The duration between symptom onset and hospital admission (day)			
More than 1	362 (87.6)	121 (33.4)	1
Less/equal 1	51 (12.4)	24 (47.1)	1.77 (0.98,3.20) ^NS^

*NS* not significant

*Significant at the level of P < 0.05

Regarding clinical symptoms at admission, 37% of patients had a temperature of more than 37.5. The most prevalent symptoms were cough (71.2%), breath shortness (61.2%), fever (32.2%), shiver (29%), and bruising (28.4%). An abnormal computed tomography was found in 94.5%. In univariate analysis, cough, shortness of breath, abdominal pain, lack of appetite, and loss of consciousness were related to the disease severity among COVID-19 patients. Shortness of breath increased the odds of severe COVID-19 (OR 1.93). The odds of severe COVID-19 was significantly greater in patients with abdominal pain (OR 7.7), seizure (OR 27.1), and loss of consciousness (OR 12.4) (Table 2). Among all patients, 1.4% had a runny nose, and 1.7% had no sign.

**Table 2:** Clinical characteristics and signs of severe COVID-19 patients in the southeast of Iran

Clinical characteristics at admission*	Total	Severe	OR (95% CI)
	N (%)	N (%)**	
Temperature			
Less/equal 37.5	257 (63)	88 (34.2)	1
37.5–38.5	107 (26.2)	38 (35.5)	1.06 (0.66,1.70)^NS^
More than 38.5	44 (10.8)	17 (38.6)	1.21 (0.62,2.34) ^NS^
Shiver			
No	294 (71)	106 (36.1)	1
Yes	119 (29)	39 (32.8)	0.86 (0.55,1.36) ^NS^
Fever			
No	320 (77.5)	112 (35.0)	1
Yes	93 (22.5)	33 (35.5)	0.85 (0.35,1.73) ^NS^
Cough			
No	119 (28.8)	54 (45.4)	1
Yes	294 (71.2)	91 (31.0)	0.54 (0.35,0.84)*
Shortness of breath			
No	160 (38.7)	42 (26.3)	1
Yes	253 (61.2)	103 (40.7)	1.93 (1.25,2.97)*
General weakness			
No	348 (84.22)	117 (33.6)	1
Yes	65 (15.8)	28 (43.1)	1.49 (0.87,2.56) ^NS^
Bruising			
No	296 (71.6)	113 (38.2)	1
Yes	117 (28.4)	32 (27.4)	0.61 (0.38,1.08)
Sore throat			
No	388 (93.9)	140 (36.1)	1
Yes	25 (6.1)	5 (20.0)	0.44 (0.16,1.21) ^NS^
Diarrhea			
No	392 (95)	142 (36.2)	1
Yes	21 (5)	3 (14.3)	0.29 (0.09,1.01) ^NS^
Nausea			
No	345 (83.5)	123 (35.7)	1
Yes	68 (16.5)	22 (32.4)	0.86 (0.50,1.50) ^NS^
Headache			
No	344 (83.3)	126 (36.6)	1
Yes	69 (16.7)	19 (27.5)	0.66 (0.37,1.16)^NS^
Chest pain			
No	401 (97)	141 (35.2)	1
Yes	12 (3)	4 (33.3)	0.92 (0.27,3.12) ^NS^
Abdominal pain			
No	403 (97.5)	137 (34.0)	1
Yes	10 (2.5)	8 (80.0)	7.77 (1.63,37.08)*
Joint pain			
No	393 (95.1)	141 (35.9)	1
Yes	20 (4.9)	4 (20.0)	0.45 (0.15,1.36) ^NS^
Tachypnea			
No	408 (98.8)	141 (34.6)	1
Yes	5 (1.2)	4 (80.0)	7.57 (0.84,68.41) ^NS^
Abnormal lung sounds			
No	369 (89.3)	129 (35.0)	1
Yes	44 (10.7)	16 (36.4)	1.06 (0.56,2.04) ^NS^
Abnormal CT			
No	23 (5.5)	6 (26.1)	1
Yes	390 (94.5)	139 (35.6)	1.57 (0.61,4.07) ^NS^
Seizure			
No	410 (99.2)	143 (34.9)	1
Yes	3 (0.8)	2 (66.7)	3.73 (0.34,41.54) ^NS^
Lack of appetite			
No	361 (87.4)	120 (33.2)	1
Yes	52 (12.6)	25 (48.1)	1.86 (1.04,3.34)*
Dizziness			
No	395 (95.6)	140 (35.4)	1
Yes	18 (4.4)	5 (27.8)	0.70 (0.24,2.01) ^NS^
Loss of consciousness			
No	386 (93.4)	122 (31.6)	1
Yes	27 (6.6)	23 (85.2)	12.44 (4.21,36.76)*
Complications created during treatment*	Total	Severe	OR (95% CI)
	N (%)	N (%)	
Sepsis			
No	387 (93.6)	121 (31.3)	1
Yes	26 (6.4)	24 (92.3)	26.38 (6.14,113.41)*
Respiratory failure			
No	378 (91.5)	112 (29.6)	1
Yes	35 (8.5)	33 (94.3)	39.19 (9.24,166.10)*
Coagulopathy			
No	409 (99)	142 (34.7)	1
Yes	4 (1)	3 (75.0)	5.64 (0.58,54.73)^NS^
Acute respiratory distress syndrome			
No	397 (96.1)	130 (32.7)	1
Yes	16 (3.9)	15 (93.8)	30.81 (4.03,235.76)*
Heart failure			
No	403 (97.5)	136 (33.7)	1
Yes	10 (2.5)	9 (90.0)	24.2 (3.33,249.09)*
Acute heart injury			
No	406 (98.3)	139 (34.2)	1
Yes	7 (1.7)	6 (85.7)	11.52 (1.37,96.68)*
Acute kidney injury			
No	393 (95.1)	128 (32.6)	1
Yes	20 (4.9)	17 (85.0)	11.73 (3.38,40.76)*
Acute liver injury			
No	410 (99.2)	143 (34.9)	1
Yes	3 (0.8)	2 (66.7)	3.73 (0.34,41.54)*
Acidosis			
No	406 (98.3)	139 (34.2)	1
Yes	7 (1.7)	6 (85.7)	11.52 (1.37,96.68)*

*NS* not significant

*Significant at the level of P < 0.05

**Percentage calculated by row

In univariate analysis, sepsis, respiratory failure, acute respiratory distress, heart failure, acute kidney and liver injury were related to the severity of disease among COVID-19 patients. The odds of severe COVID-19 was significantly greater in patients with sepsis (OR 26.3), respiratory failure (OR 39.1), acute respiratory distress syndrome (OR 30.81), heart failure (OR 24.2), acute heart injury (OR 11.5), acute kidney injury (OR 11.7), liver injury (OR 3.7) and acidosis (OR 11.5) (Table 2).

The most prevalent underlying disease among COVID-19 patients were hypertension (23.5%), diabetes (17.2%), cardiovascular disease (13.1%), chronic pulmonary disease (9.5%), and asthma (5.1%). Underlying disease, including cardiovascular disease (OR 2.43), liver disease (OR 5.74), chronic lung disease (OR 3.34), hypertension (OR 1.98), and rheumatologic disease (OR 5.8), increased the odds of severity among COVID-19 patients (Table 3). Congestive heart disease, organ transplant, and malignancy were reported in only 2 (0.5%) patients.

**Table 3:** Distribution of underlying disease among severe COVID-19 patients in the southeast Iran

Underlying disease	Total	Severe	OR (95% CI)
	N (%)	N (%)**	
Dialysis			
No	401 (94.8)	140 (34.9)	1
Yes	12 (5.2)	5 (41.7)	1.33 (0.42,4.27)^NS^
Cardiovascular disease			
No	359 (86.9)	116 (32.3)	1
Yes	54 (13.1)	29 (53.7)	2.43 (1.36,4.34)*
Diabetes			
No	342 (82.8)	114 (33.3)	1
Yes	71 (17.2)	31 (43.7)	1.55 (0.92,2.61) ^NS^
Liver disease			
No	405 (98)	139 (34.3)	1
Yes	8 (2)	6 (75.0)	5.74 (1.14,28.82)*
Chronic kidney disease			
No	400 (96.8)	138 (34.5)	1
Yes	13 (3.2)	7 (53.8)	2.22 (0.73,6.72) ^NS^
Chronic neurological disease			
No	404 (97.8)	143 (35.4)	1
Yes	9 (2.2)	2 (22.2)	0.52 (0.11,2.54) ^NS^
Chronic pulmonary disease			
No	374 (90.5)	121 (32.4)	1
Yes	39 (9.5)	24 (61.5)	3.34 (1.69,6.61)*
Malignant Disease			
No	411 (99.5)	144 (35.0)	1
Yes	2 (0.5)	1 (50.0)	1.85 (0.12,29.86) ^NS^
Hypertension			
No	316 (76.5)	99 (31.3)	1
Yes	97 (23.5)	46 (47.4)	1.98 (1.24,3.14)*
Cerebrovascular disease			
No	404 (97.8)	140 (34.7)	1
Yes	9 (2.2)	5 (55.6)	2.36 (0.62,8.92) ^NS^
Chronic blood disease			
No	405 (98)	142 (35.1)	1
Yes	8 (2)	3 (37.5)	1.11 (0.26,4.72) ^NS^
Asthma			
No	392 (94.9)	136 (34.7)	1
Yes	21 (5.1)	9 (42.9)	1.41 (0.58,3.43) ^NS^
Congestive heart disease			
No	411 (99.5)	143 (34.8)	
Yes	2 (0.5)	2 (100.0)	
Rheumatologic disease			
No	401 (97.1)	136 (33.9)	1
Yes	12 (2.9)	9 (75.0)	5.85 (1.56,21.95)*
Organ transplant			
No	411 (99.5)	143 (34.8)	
Yes	2 (0.5)	2 (100.0)	
Defect immune system			
No	400 (96.8)	140 (35.0)	1
Yes	13 (3.2)	5 (38.5)	1.16 (0.37,3.62) ^NS^

*NS* no significant

*Significant at the level of P < 0.05

**Percentage calculated by row

Among all patients, 37% had an oxygen saturation of more than 0.93, 11.4% were eventually admitted to the ICU, and 8.7% received mechanical ventilation. According to oxygen therapy, 90.6% received high flow nasal cannula (HFNC) and 8.2% invasive mechanical ventilation. The odds of severe disease increased significantly in patients admitted to ICU (OR 27.8) and mechanically ventilated patients (OR 84.9). Oxygen saturation of more than 0.93 could prevent the disease severity by 70%. The mean heart rate and respiratory rate were significantly different between the severe and non-severe patients (P < 0.001) (Table 4). About 36 (94.7%) of the severe patients were intubated. Most patients took antibiotics (91.5%) and antiviral drugs (97.3%) but were not related to severity among COVID-19 patients (P > 0.05).

**Table 4:** Distribution of oxygen therapy and vital sign among severe COVID-19 patients in the southeast Iran

	Total	Severe	OR (95% CI)
	N (%)	N (%)**	
High flow nasal cannula			
No	39 (90.5)	37 (94.9)	1
Yes	374 (9.5)	108 (28.9)	0.02 (0.005,0.093)*
Non-Invasive mechanical ventilation			
No	405 (98)	137 (33.8)	
Yes	8 (2)	8 (100.0)	
Invasive mechanical ventilation			
No	379 (91.7)	112 (29.6)	1
Yes	34 (8.3)	33 (97.1)	78.67 (10.63,582.25)*
ICU admission			
No	366 (88.6)	102 (27.9)	1
Yes	47 (11.4)	43 (91.5)	27.82 (9.74,79.49)*
Intubation			
No	375 (90.8)	109 (29.1)	1
Yes	38 (9.2)	36 (94.7)	43.93 (10.40,185.63)*
Mechanical ventilation			
No	377 (91.3)	110 (29.2)	1
Yes	36 (8.7)	35 (97.2)	84.96 (11.50,627.82)*
Oxygen saturation			
More than 0.93	153 (37)	30 (19.6)	1
Less/equal 0.93	258 (63)	114 (44.2)	3.25 (2.03,5.18)*

Mean ± SD	Low/Moderate	Sever/Critically sever	P
Heart rate	92.33 ± 12.94	97.75 ± 18.57	0.002
Respiratory rate	18.92 ± 4.37	22.19 ± 6.53	< 0.001
Systolic blood pressure	115.95 ± 16.35	118.36 ± 19.21	0.182^NS^
Diastolic blood pressure	73.18 ± 11.14	74.57 ± 11.46	0.234^NS^

*NS* no significant

*Significant at the level of P < 0.05

**Percentage calculated by row

Multivariate analysis was performed on all variables found significant by univariate analysis in Tables 1, 2 and 3. Multivariate regression showed increased odds of the severity for COVID-19 was associated with older age (OR 2.27), substance abuse (OR 2.49), having one underlying disease (OR 1.52), having two underlying diseases (OR 2.31), having three or more underlying diseases (OR 2.60) (Table 5).

**Table 5:** Multivariate logistic regression for predictors of severity among COVID-19 patients in the southeast of Iran

Independent variables	B	S.E of Beta	P	OR	OR (95% CI)	
Having underlying disease***					Lower	Upper
No**				1		
One	0.841	0.265	0.114	1.521	0.904	2.558
Two	0.957	0.339	0.013	2.319	1.193	4.504
Three or more	0.913	0.397	0.016	2.604	1.197	5.665
Substance abuse						
No**				1		
Yes	0.913	0.397	0.022	2.493	1.144	5.432
Age						
> 50				1		
≤ 50**	0.823	0.241	0.001	2.277	1.419	3.655

*Variables for Table 4 not included in the model

**Reference group

***Having underlying disease according to Table 3

## Discussion

The current study investigated the clinical characteristics of patients with COVID-19 and potential risk factors for severity. Univariate analysis revealed that age, substance abuse, traveling to high-risk areas, means of transportation to a medical center, cough, shortness of breath, abdominal pain, lack of appetite, loss of consciousness, sepsis, respiratory failure, acute respiratory distress syndrome were associated with severity in COVID-19 patients. Also, heart failure, acute heart injury, acute kidney injury, acute liver injury, acidosis, cardiovascular disease, liver disease, chronic pulmonary disease, hypertension, rheumatologic disease, high flow nasal cannula oxygen therapy, invasive mechanical ventilation, ICU admission, intubation, mechanical ventilation, oxygen saturation, heart rate, and respiratory rate were associated with severity in COVID-19 patients. Multivariate analysis revealed that age, substance abuse, and underlying disease were predictors of severity in COVID-19 patients.

Among the 413 patients in this study, 145 (35.10%) were in severe condition, and older patients were at higher risk of severe disease. Inadequate immune response in older patients leads to more complicated and critical conditions such as ARDS, severe clinical manifestations, and longer disease duration [18, 19]. Other studies have reported a lower prevalence of disease severity, including 17.6% and 25.6% [20, 21], which is expected based on various definitions.

In the current study, substance abuse was a risk factor for COVID-19 severity, resulting from direct damage to the respiratory system, modulating brain and immune functions [22]. Severe disease was also associated with travel to high-risk areas. This finding highlights the importance of social distancing on viral inocula reduction [23], leading to less severe disease [24]. Exposure to confirmed cases and recent travel to the epidemic area are the host risk factors for severe COVID-19 [25].

In terms of signs, this study showed that cough, shortness of breath, abdominal pain, losing appetite, heart rate, and the number of breathing were associated with severity in COVID-19 patients. Gastrointestinal symptoms are more prevalent in severe COVID-19 patients, so 100% of these patients experience a lack of appetite. The increased viral load and the spread of the virus in the gastrointestinal tract of COVID-19 patients can lead to the severity of the disease [26]. Other studies have shown that cough, shortness of breath, and abdominal pain are common in patients with severe COVID-19 [27–29]. Furthermore, high respiratory and heart rates were the risk factors for the severity in COVID-19 patients [30].

The present study showed a decreased level of consciousness, oxygen requirement at hospitalization, ICU admission, invasive mechanical ventilation, intubation, and oxygen saturation were risk factors for severity in COVID-19 patients. Previous studies have shown that only severe patients need to be admitted to the ICU, and oxygen saturation is lower in severe patients. In addition, unconsciousness, shortness of breath, oxygen therapy, invasive and non-invasive mechanical ventilation were also more common in severe patients [31–34].

In the current study, complications during treatment, sepsis, respiratory failure, acute respiratory distress syndrome, heart failure, acute heart injury, acute kidney injury, and acidosis were risk factors for severity in COVID-19 patients. The cause of respiratory failure in patients with COVID-19 was impaired immune function [35]. Uncontrolled viral infection causes macrophage penetration and further damage to the lungs [36]. Serious complications such as acute respiratory distress syndrome, acute cardiac injury, acute kidney injury, and shock can occur in patients with severe COVID-19. Acute respiratory distress syndrome and acute cardiac injury are the most important barriers to treating COVID-19 patients [37]. Cardiac injury is associated with the severity of disease in patients with COVID-19 [38]. The SARS-CoV-2 affects the cardiovascular system through angiotensin-converting enzyme 2 (ACE2) and causes myocardial injury and heart failure [39, 40]. Acute kidney injury (AKI) is an indicator of disease severity. Volume depletion may be the cause of acute kidney injury [41]. The prevalence of liver dysfunction and liver injury is higher in patients with severe COVID-19. Liver impairment may be caused directly by a viral infection of the liver cells or by hepatotoxicity caused by drugs and immune-mediated inflammation [42].

In terms of underlying health conditions, cardiovascular disease, liver disease, chronic pulmonary disease, hypertension, and rheumatologic disease were risk factors for severe COVID-19 in this study. Underlying health conditions are responsible for 20% of severe COVID-19 worldwide [43]. Severe COVID-19 patients experience a higher incidence of comorbidity, a risk factor for the severity of COVID-19 pneumonia [44, 45]. Patients with established cardiovascular disease exhibited a greater angiotensin-converting enzyme 2 (ACE2) expression and probably experienced a worse condition following SARS-CoV-2 infection [46, 47]. Patients with a history of hypertension are more likely to develop severe COVID-19 as hypertension is a predictor of severe pneumonia [48, 49]. There is evidence that antihypertensive drugs prevent patients with comorbid hypertension from severe pneumonia. Chronic respiratory conditions are also associated with the severity of COVID-19 [50]. Elevated liver biochemical indicators are common in COVID‐19, resulting in severe COVID‐19 [51, 52]. Patients with rheumatic diseases are at high risk for COVID-19 infection due to their immune conditions [53]. In addition, chronic inflammatory rheumatic patients with autoimmune or immune-mediated diseases are at risk of severe COVID-19 [54].

## Conclusions

COVID-19 was more severe in older patients, patients with a history of substance abuse, and patients with underlying disease. Understanding the factors affecting the disease severity can help the clinical management of COVID-19, especially in less privileged areas where fewer resources are available.

One of the strengths of the present study is that the patients were selected from a less privileged area which can draw the attention of health policymakers to this area. Another strength of the study is the multi-center nature of the survey, representing the population. This study had some limitations: First, the small sample size, leading to insignificant statistical results. Therefore, the results of this study should be interpreted with caution. Further studies are needed to investigate potential risk factors of severity in patients with COVID-19 with large sample size. Second, the information was collected as a self-report in some cases, leading to recall bias.

## Data Availability

The data used in this study are available upon reasonable request from the corresponding author.

## Abbreviations

COVID-19: Coronavirus disease
OR: Odds ratio
WHO: World Health Organization
ICU: Intensive care unit
RT-PCR: Real-time reverse-transcription-polymerase-chain-reaction
SD: Standard deviation
HFNC: High flow nasal cannula
ARDS: Acute respiratory distress syndrome
AKI: Acute kidney injury
ACE2: Angiotensin-converting enzyme 2

## References

[1] Lai C-C, Shih T-P, Ko W-C, Tang H-J, Hsueh P-R (2020). Severe acute respiratory syndrome coronavirus 2 (SARS-CoV-2) and corona virus disease-2019 (COVID-19): the epidemic and the challenges. Int J Antimicrob Agents.

[2] WHO. Novel Coronavirus – China 2020. https://www.who.int/csr/don/12-january-2020-novel-coronavirus-china/en/.

[3] WHO. WHO Coronavirus Disease (COVID-19) Dashboard 2020. https://www.worldometers.info/coronavirus/#countries.

[4] Chen N, Zhou M, Dong X, Qu J, Gong F, Han Y (2020). Epidemiological and clinical characteristics of 99 cases of 2019 novel coronavirus pneumonia in Wuhan, China: a descriptive study. Lancet.

[5] Huang C, Wang Y, Li X, Ren L, Zhao J, Hu Y (2020). Clinical features of patients infected with 2019 novel coronavirus in Wuhan, China. Lancet.

[6] Wang C, Horby PW, Hayden FG, Gao GF (2020). A novel coronavirus outbreak of global health concern. Lancet.

[7] Shim E, Tariq A, Choi W, Lee Y, Chowell G (2020). Transmission potential and severity of COVID-19 in South Korea. Int J Infect Dis.

[8] Huang R, Zhu L, Xue L, Liu L, Yan X, Wang J (2020). Clinical findings of patients with coronavirus disease 2019 in Jiangsu province, China: a retrospective, multi-center study. PLoS Neglect Trop Dis..

[9] Wang D, Hu B, Hu C, Zhu F, Liu X, Zhang J (2020). Clinical characteristics of 138 hospitalized patients with 2019 novel coronavirus–infected pneumonia in Wuhan, China. JAMA.

[10] Yang J, Zheng Y, Gou X, Pu K, Chen Z, Guo Q (2020). Prevalence of comorbidities in the novel Wuhan coronavirus (COVID-19) infection: a systematic review and meta-analysis. Int J Infect Dis.

[11] Livingston E, Bucher K (2020). Coronavirus disease 2019 (COVID-19) in Italy. JAMA.

[12] Fang L, Gao P, Bao H, Tang X, Wang B, Feng Y (2018). Chronic obstructive pulmonary disease in China: a nationwide prevalence study. Lancet Respir Med.

[13] Ruan Q, Yang K, Wang W, Jiang L, Song J (2020). Clinical predictors of mortality due to COVID-19 based on an analysis of data of 150 patients from Wuhan, China. Intensive Care Med.

[14] Zhu Y, Duan M-J, Dijk HH, Freriks RD, Dekker LH, Mierau JO (2021). Association between socioeconomic status and self-reported, tested and diagnosed COVID-19 status during the first wave in the Northern Netherlands: a general population-based cohort from 49 474 adults. BMJ Open.

[15] Azar KM, Shen Z, Romanelli RJ, Lockhart SH, Smits K, Robinson S (2020). Disparities in outcomes among COVID-19 patients in a large health care system in California: study estimates the COVID-19 infection fatality rate at the US county level. Health Aff.

[16] Afrakhteh H (2006). The problems of regional development and border cities: A case study of Zahedan. Iran Cities.

[17] World Health Organization (2020). Clinical management of COVID-19: interim guidance, 27 May 2020.

[18] Wu C, Chen X, Cai Y, Zhou X, Xu S, Huang H (2020). Risk factors associated with acute respiratory distress syndrome and death in patients with coronavirus disease 2019 pneumonia in Wuhan, China. JAMA Internal Med.

[19] Liu Y, Mao B, Liang S, Yang J-W, Lu H-W, Chai Y-H (2020). Association between ages and clinical characteristics and outcomes of coronavirus disease 2019. Eur Respir J.

[20] Tian S, Hu N, Lou J, Chen K, Kang X, Xiang Z (2020). Characteristics of COVID-19 infection in Beijing. J Infect.

[21] Fu L, Wang B, Yuan T, Chen X, Ao Y, Fitzpatrick T (2019). Clinical characteristics of coronavirus disease 2019 (COVID-19) in China: a systematic review and meta-analysis. J Infect.

[22] Wei Y, Shah R (2020). Substance use disorder in the COVID-19 pandemic: a systematic review of vulnerabilities and complications. Pharmaceuticals.

[23] Dalton CB, Corbett SJ, Katelaris AL (2020). Pre-emptive low cost social distancing and enhanced hygiene implemented before local COVID-19 transmission could decrease the number and severity of cases. Med J Aust.

[24] Yu X, Sun S, Shi Y, Wang H, Zhao R, Sheng J (2020). SARS-CoV-2 viral load in sputum correlates with risk of COVID-19 progression. Crit Care.

[25] Shi Y, Yu X, Zhao H, Wang H, Zhao R, Sheng J (2020). Host susceptibility to severe COVID-19 and establishment of a host risk score: findings of 487 cases outside Wuhan. Crit Care.

[26] Pan L, Mu M, Yang P, Sun Y, Wang R, Yan J (2020). Clinical characteristics of COVID-19 patients with digestive symptoms in Hubei, China: a descriptive, cross-sectional, multicenter study. Am J Gastroenterol.

[27] Zheng Z, Peng F, Xu B, Zhao J, Liu H, Peng J (2020). Risk factors of critical & mortal COVID-19 cases: A systematic literature review and meta-analysis. J Infect.

[28] Mo P, Xing Y, Xiao Y, Deng L, Zhao Q, Wang H (2020). Clinical characteristics of refractory COVID-19 pneumonia in Wuhan, China. Clin Infect Dis.

[29] Li X, Xu S, Yu M, Wang K, Tao Y, Zhou Y (2020). Risk factors for severity and mortality in adult COVID-19 inpatients in Wuhan. J Allergy Clin Immunol.

[30] Zhang J, Yu M, Tong S, Liu L-Y, Tang L-V (2020). Predictive factors for disease progression in hospitalized patients with coronavirus disease 2019 in Wuhan, China. J Clin Virol.

[31] Liu Y, Yan L-M, Wan L, Xiang T-X, Le A, Liu J-M (2020). Viral dynamics in mild and severe cases of COVID-19. Lancet Infect Dis.

[32] Wei Y-Y, Wang R-R, Zhang D-W, Tu Y-H, Chen C-S, Ji S (2020). Risk factors for severe COVID-19: Evidence from 167 hospitalized patients in Anhui, China. J Infect.

[33] Bhargava A, Fukushima EA, Levine M, Zhao W, Tanveer F, Szpunar SM (2020). Predictors for severe COVID-19 infection. Clin Infect Dis.

[34] Zhang J, Wang X, Jia X, Li J, Hu K, Chen G (2020). Risk factors for disease severity, unimprovement, and mortality of COVID-19 patients in Wuhan, China. Clin Microbiol Infect.

[35] Giamarellos-Bourboulis EJ, Netea MG, Rovina N, Akinosoglou K, Antoniadou A, Antonakos N (2020). Complex immune dysregulation in COVID-19 patients with severe respiratory failure. Cell Host Microbe.

[36] Li H, Liu L, Zhang D, Xu J, Dai H, Tang N (2020). SARS-CoV-2 and viral sepsis: observations and hypotheses. Lancet.

[37] Hu Y, Sun J, Dai Z, Deng H, Li X, Huang Q (2019). Prevalence and severity of corona virus disease 2019 (COVID-19): A systematic review and meta-analysis. J Clin Virol.

[38] Santoso A, Pranata R, Wibowo A, Al-Farabi MJ, Huang I, Antariksa B (2020). Cardiac injury is associated with mortality and critically ill pneumonia in COVID-19: a meta-analysis. Am J Emerg Med.

[39] Long B, Brady WJ, Koyfman A, Gottlieb M (2020). Cardiovascular complications in COVID-19. Am J Emerg Med.

[40] Clerkin KJ, Fried JA, Raikhelkar J, Sayer G, Griffin JM, Masoumi A (2020). COVID-19 and cardiovascular disease. Circulation.

[41] Ronco C, Reis T, Husain-Syed F (2020). Management of acute kidney injury in patients with COVID-19. Lancet Respir Med.

[42] Zhang C, Shi L, Wang F-S (2020). Liver injury in COVID-19: management and challenges. Lancet Gastroenterol Hepatol.

[43] Clark A, Jit M, Warren-Gash C, Guthrie B, Wang HH, Mercer SW (2020). Global, regional, and national estimates of the population at increased risk of severe COVID-19 due to underlying health conditions in 2020: a modelling study. Lancet Glob Health.

[44] Li K, Wu J, Wu F, Guo D, Chen L, Fang Z (2020). The clinical and chest CT features associated with severe and critical COVID-19 pneumonia. Investig Radiol..

[45] Yin Q, Fu Z, Xie J, Yang J, Li F, Zhu W, et al. Analysis of Risk Factors of Severe COVID-19 Patients. 2020.

[46] Chen L, Li X, Chen M, Feng Y, Xiong C (2020). The ACE2 expression in human heart indicates new potential mechanism of heart injury among patients infected with SARS-CoV-2. Cardiovasc Res.

[47] Li B, Yang J, Zhao F, Zhi L, Wang X, Liu L (2020). Prevalence and impact of cardiovascular metabolic diseases on COVID-19 in China. Clin Res Cardiol.

[48] Liu S, Luo H, Wang Y, Cuevas LE, Wang D, Ju S (2020). Clinical characteristics and risk factors of patients with severe COVID-19 in Jiangsu province, China: a retrospective multicentre cohort study. BMC Infect Dis.

[49] Liu X, Zhou H, Zhou Y, Wu X, Zhao Y, Lu Y (2020). Risk factors associated with disease severity and length of hospital stay in COVID-19 patients. J Infect.

[50] Izcovich A, Ragusa M, Tortosa F, Lavena Marzio MA, Agnoletti C, Bengolea A (2020). Prognostic factors for severity and mortality in patients infected with COVID-19: a systematic review. PLoS ONE.

[51] Wu Y, Li H, Guo X, Yoshida EM, Mendez-Sanchez N, Sandri GBL (2020). Incidence, risk factors, and prognosis of abnormal liver biochemical tests in COVID-19 patients: a systematic review and meta-analysis. Hepatol Int.

[52] Kulkarni AV, Kumar P, Tevethia HV, Premkumar M, Arab JP, Candia R (2020). Systematic review with meta-analysis: liver manifestations and outcomes in COVID-19. Alimentary Pharmacol Therap..

[53] Gianfrancesco MA, Hyrich KL, Gossec L, Strangfeld A, Carmona L, Mateus EF (2020). Rheumatic disease and COVID-19: initial data from the COVID-19 global rheumatology alliance provider registries. Lancet Rheumatol.

[54] Pablos J, Galindo M, Carmona L, Retuerto M, Lledó A, Blanco R (2020). Clinical outcomes of patients with COVID-19 and chronic inflammatory and autoimmune rheumatic diseases: a multicentric matched-cohort study. Ann Rheum Dis.

